# Multi-strategy modified sparrow search algorithm for hyperparameter optimization in arbitrage prediction models

**DOI:** 10.1371/journal.pone.0303688

**Published:** 2024-05-15

**Authors:** Shenjie Cheng, Panke Qin, Baoyun Lu, Jinxia Yu, Yongli Tang, Zeliang Zeng, Sensen Tu, Haoran Qi, Bo Ye, Zhongqi Cai

**Affiliations:** 1 School of Software, Henan Polytechnic University, Jiaozuo, China; 2 Hebi National Optoelectronic Technology Co, Ltd, Hebi, China; University of Barcelona: Universitat de Barcelona, SPAIN

## Abstract

Deep learning models struggle to effectively capture data features and make accurate predictions because of the strong non-linear characteristics of arbitrage data. Therefore, to fully exploit the model performance, researchers have focused on network structure and hyperparameter selection using various swarm intelligence algorithms for optimization. Sparrow Search Algorithm (SSA), a classic heuristic method that simulates the sparrows’ foraging and anti-predatory behavior, has demonstrated excellent performance in various optimization problems. Hence, in this study, the Multi-Strategy Modified Sparrow Search Algorithm (MSMSSA) is applied to the Long Short-Term Memory (LSTM) network to construct an arbitrage spread prediction model (MSMSSA-LSTM). In the modified algorithm, the good point set theory, the proportion-adaptive strategy, and the improved location update method are introduced to further enhance the spatial exploration capability of the sparrow. The proposed model was evaluated using the real spread data of rebar and hot coil futures in the Chinese futures market. The obtained results showed that the mean absolute percentage error, root mean square error, and mean absolute error of the proposed model had decreased by a maximum of 58.5%, 65.2%, and 67.6% compared to several classical models. The model has high accuracy in predicting arbitrage spreads, which can provide some reference for investors.

## 1 Introduction

The futures market plays a significant contribution to stabilizing commodity prices and promoting capital flow. Among them, cross-variety arbitrage trading in futures has attracted widespread attention from investors due to its low cost, small risk, and relatively stable returns. Its essence is spread arbitrage, obtaining returns based on the regression of spreads between futures contracts with stronger correlation. Therefore, in order to assist investors in formulating more scientific trading strategies to obtain substantial profits, it is extremely important to accurately predict the trend of arbitrage spreads between futures contracts. However, the market is dynamic and volatile, and the pricing of contracts is often based on a variety of complex dynamic conditions [1]. These factors make the prediction of arbitrage spread trends a significant challenge.

The traditional solution to the problem of arbitrage spread trend prediction is to utilize time series models, such as the autoregressive conditional heteroskedasticity (ARCH) model, the autoregressive moving average (ARMA) model, the autoregressive integrated moving average (ARIMA) model, etc [2–5]. Although these models can fit the current data well, the prediction effect is not ideal when facing out-of-sample data.

In recent years, with the development and perfection of artificial intelligence technology, numerous machine learning models have been widely used in the field of financial time series prediction due to their outstanding fitting ability. Tay and Cao [6] used support vector machines (SVM) to predict the closing price of the Standard&Poor 500 stock index futures. The experimental results show that this method is more effective than the back propagation (BP) neural network model. Nayak et al. [7] combined the artificial chemical reaction optimization (ACRO) algorithm with the multilayer perceptron (MLP) to construct an artificial chemical reaction neural network (ACRNN) for predicting stock market indices. Li [8] achieved the prediction of the settlement price of China’s stock index futures through empirical mode decomposition (EMD) and radial basis function (RBF) neural networks. Compared with the traditional time series model, the aforementioned methods have achieved a significant improvement in prediction accuracy. Meanwhile, with the advent of the big data era, scholars are conducting more and more research on deep neural network (DNN) models [9, 10]. Hu [11] applied a convolutional neural network (CNN) to predict stock prices. However, since CNNs are better at handling image problems, their accuracy is lower when facing time series prediction. Berradi and Lazaar [12] predicted the stock price of Total Maroc from the Casablanca Stock Exchange through a recurrent neural network (RNN) and used principal component analysis (PCA) for dimensionality reduction, ultimately obtaining superior prediction results. It is noteworthy that despite their effectiveness, there are still some issues with the RNN model. Hochreiter and Schmidhuber [13] improved its unit structure and proposed the long short-term memory (LSTM) network model, which effectively addressed their shortcomings such as insufficient long-term memory capacity through the design of gate structures. Numerous studies have shown that LSTM networks are able to discover long-term dependencies in sequence information well, and are therefore widely used in the field of financial forecasting [14–17].

To further enhance the predictive performance of the LSTM model, it is necessary to optimize its hyperparameters. However, there is no clear function relationship between model performance and hyperparameters. Therefore, in practical applications, researchers often determine the optimal values of hyperparameters based on their own experience, existing research, and abundant experimental results. This approach not only results in a significant waste of manpower and computational resources, but also introduces subjective factors that make it difficult to ensure the optimality of the model. When the hyperparameter space is more complex, the entire optimization process can be extremely time-consuming and inefficient. Therefore, finding the optimal combination of hyperparameters in neural network models has also become a challenging task.

In addition, in the futures market, arbitrage methods are mainly divided into two categories: mean reversion arbitrage methods and neural network arbitrage methods [18]. The mean reversion method uses financial time series analysis methods to study the long-term relationships that exist between futures, so as to design arbitrage strategies. For example, Liu and Lan [19] constructed cointegration regression and vector error correction model to analyze the monthly average closing price of polyvinyl chloride futures contracts. The results found that there is a long-term equilibrium and short-term deviation relationship of the contract spread, confirming the existence of intertemporal arbitrage opportunities in this contract. Liu [20] applied the cointegration method to empirically analyze the prices of hogs, corn, and soybean meal. It was found that there is a long-term mean relationship between the three, and the trading simulation also confirmed the possibility of profit. The neural network arbitrage method utilizes neural network models to predict spreads and formulate arbitrage strategies. At present, the existing domestic research is mainly based on the mean reversion principle of spreads. There are fewer studies on using neural network for arbitrage, and most of them have the disadvantages of single model and low prediction accuracy [21, 22]. Therefore, to develop scientific and efficient arbitrage strategies, it is extremely important to accurately predict the arbitrage spreads between futures.

Given the above reasons, this article proposes an arbitrage spread prediction model based on multi-strategy modified SSA-optimized LSTM (MSMSSA-LSTM) by combining the sparrow search algorithm [23] (SSA) with the LSTM network. This research have constructed a regression model with high prediction accuracy by using MSMSSA to match the futures data features and LSTM neural network topology structure. On this basis, this paper conducts an empirical analysis using the spread dataset of rebar and hot coil futures in the Chinese futures market.

The main contributions of this article can be summarized as follows:

- This research has constructed the MSMSSA-LSTM model by improving the sparrow search algorithm to achieve the trend prediction of arbitrage spread between futures.
- To verify the effectiveness of the MSMSSA-LSTM model, this research selected the MLP model, RNN model, LSTM model, gated recurrent unit (GRU) model, and LSTM model optimized by traditional sparrow search algorithm (SSA-LSTM) as comparative experiments. The experimental results indicate that the MSMSSA-LSTM model has high prediction accuracy and is more suitable for predicting the trend of arbitrage spread between futures.

The remaining chapters of this paper are organized as follows. Section 2 introduces the hyperparameter optimization problem in the LSTM network model and its solutions. Section 3 analyzes some literature on optimizing LSTM models using metaheuristic algorithms. Section 4 describes the sparrow search algorithm and its improvement strategies, and construct the MSMSSA-LSTM model. Section 5 validates the effectiveness of the MSMSSA-LSTM model in predicting arbitrage spread trends through comparative experiments. Finally, the conclusion is given in Section 6.

## 2 Problem description

In neural networks, there are many parameters that need to be set manually which are hyperparameters. The selection of suitable hyperparameter values plays a crucial role in the final prediction performance of the model. Therefore, how to choose appropriate hyperparameters based on the characteristics of the data has always been a widely studied topic.

This section first described the hyperparameter optimization problem using mathematical formulas. Secondly, several existing solutions to this problem and their respective drawbacks were given. Finally, a new solution was proposed.

The following equation can be used to mathematically represent the hyperparameter optimization problem in an LSTM network model:

$$\{\begin{array}{lll} \min & MSE=\frac{1}{n}\sum_{j=1}^{n}{\left({\hat{y}}_{j}(X)-y_{j}\right)}^{2} & \\ s.t. & x_{i}\in [x_{is},x_{ib}] & \left(i=1,2,\cdots ,d\right)\\ & X={\left[x_{1},x_{2},\cdots ,x_{d}\right]}^{T} & \end{array}$$ (1)

In the equation, *d* represents the number of hyperparameters that need to be optimized in the LSTM network. [*x*_*ix*_,*x*_*ib*_] denotes the range of values for the i-th hyperparameter, with a lower limit of *x*_*is*_ and an upper limit of *x*_*ib*_. *X* indicates one combination of values taken by the *d* hyperparameters. The total number of samples in the validation set is represented by *n*. *y*_*j*_ stands for the actual value of the j-th sample. When the hyperparameters are *X*, the model’s predicted value for the j-th specimen is ${\hat{y}}_{j}(X)$. The optimization goal of the LSTM network model is to find an optimal hyperparameter combination *X*, which minimizes the mean square error *MSE* on the validation set.

The traditional hyperparameter optimization techniques include grid search, random search, and Bayesian optimization. Among them, grid search determines the optimal solution by exhaustively traversing all combinations of hyperparameters. Random search searches for the global optimal solution by randomly selecting sample points. Bayesian optimization attempts to seek the optimal solution by constructing a posterior probability of the black box function output. Compared to the three, Bayesian optimization fully considers the existing hyperparameter combination information, while grid search and random search ignore this information, which can lead to serious resource waste. Although Bayesian optimization has shown good results in finding the optimal combination of hyperparameters, it still has the disadvantages of slow search speed and easy to fall into local optimal solutions. Therefore, this paper effectively solves the above problems by combining the improved SSA algorithm with the LSTM network model.

## 3 Related works

In related work, there is a lot of research on using heuristic algorithms to optimize LSTM models. Some of them are listed below.

In 2022, Drewil and Al-Bahadili [24] applied genetic algorithms to find the optimal values of window size and number of units in the LSTM network model. They selected air pollution prediction for experiments and proved that the model modified by the optimization algorithm outperformed the benchmark model. In 2023, Bacanin et al. [25] optimized the learning rate, dropout rate, number of epochs, number of layers, and number of neuron cells in each layer in the LSTM network using the improved particle swarm optimization algorithm. The experimental results of cloud load prediction show that the optimized LSTM has superior performance to other performed techniques. In 2020, Kumar and Haider [26] used the flower pollination algorithm and particle swarm optimization algorithm to optimize the time lag, number of hidden layers, number of hidden neurons, batch size, and epochs in the RNN-LSTM model, respectively. The experimental results demonstrate that the optimized model enhances performance and has higher accuracy. In 2022, Jovanovic et al. [27] applied the salp swarm algorithm with a disputation operator to optimize the learning rate, dropout rate, number of neurons in the LSTM layer, and the number of training epochs in the LSTM network. They selected the West Texas Intermediate dataset for testing. The obtained results demonstrate that the proposed model outperforms all other competitors and exhibits the best performance. In 2024, Zhang et al. [28] optimized the Bi-LSTM model by using the whale optimization algorithm with circle mapping and self-adaptive weight adjustment. The accuracy of the proposed method is proved by the plug-load electricity consumption prediction. In 2022, Bacanin et al. [29] achieved smart air quality prediction and node localization based on the Graph LSTM and the improved dragonfly optimizer algorithm. In 2023, Gülmez [30] optimized the learning rate, dropout rate, optimizer algorithm, layer existing or not existing, and number of neurons in the LSTM model using the artificial rabbit optimization algorithm. Dow Jones Index stock price data was used for testing. The results indicate that the model has certain universality and good prediction accuracy.

## 4 Methodology

Section 4.1 introduces the traditional sparrow search algorithm. Section 4.2 presents three improvement strategies and name the improved algorithm as the multi-strategy modified sparrow search algorithm (MSMSSA). Additionally, this section also provides the pseudocode of MSMSSA. Section 4.3 constructs the MSMSSA-LSTM model and introduce its structure and execution process.

### 4.1 Sparrow search algorithm

The sparrow search algorithm is a swarm intelligence optimization algorithm proposed based on the foraging and anti-predation behavior of sparrow populations. When foraging, sparrows will be divided into two types, discoverers and followers, based on the quality of the food searched. The discoverer is responsible for finding food and providing the foraging area and direction for the followers. The followers follow the discoverer to get better food. If the follower’s position is poor, it will fly to other areas to forage. In addition, when a sparrow individual discovers predators around the population, it will send out an alarm signal and move to a safe area. Sparrows in the middle of the group will randomly approach other sparrows. Once the alarm value is higher than the safety value, the discoverer will immediately lead the followers out of the danger zone and fly to other safe areas to forage.

Assuming there are n sparrows in the sparrow population and the dimension of the search space is d, then the position information of all sparrows can be regarded as an n×d matrix. The position of each sparrow can be represented as x_i,j_, where i = 1, 2, 3, …, n, j = 1, 2, 3, …, d. x_i,j_ indicates the position information of the i-th sparrow in the j-th dimension. The quality of the food searched by each sparrow is reflected by the fitness function. The fitness value of each sparrow can be expressed as F_xi_ = f([x_i,1_, x_i,2_, x_i,3_, …, x_i,d_]).

Before each iteration, sparrows are sorted based on the size of their fitness values. The top 10% to 20% of sparrows are the discoverers. The position update formula for the discoverers can be described by the following equation:

$$X_{i}^{t+1}=\{\begin{array}{ll} X_{i}^{t}\cdot \exp(-\frac{i}{\alpha \cdot T}), & R_{2}<ST\\ X_{i}^{t}+Q\cdot L, & R_{2}\geq ST \end{array}$$ (2)

In this expression, t denotes the current number of iterations and T is a constant representing the total number of iterations. X_i_ denotes the position corresponding to the sparrow whose fitness value is ranked i in the population. α is a random number between (0, 1]. Q is a random number that follows a standard normal distribution. L is a 1×d matrix with all elements being 1. R_2_ and ST indicate the alarm value and safety value, respectively, with their ranges being [0, 1] and [0.5, 1], respectively. When R_2_ < ST, it represents that no predators are detected at this time, and the discoverer can perform a wide range of searches. When R_2_ ≥ ST, it indicates that there are a large number of predators around the foraging environment at this time, and the discoverer needs to immediately lead the followers to forage in other safe areas.

The position update formula for the followers can be described by the following equation:

$$X_{i}^{t+1}=\{\begin{array}{ll} Q\cdot \exp(\frac{X_{worst}^{t}-X_{i}^{t}}{i^{2}}), & i>n/2\\ X_{p}^{t+1}+|X_{i}^{t}-X_{p}^{t+1}|\cdot A^{+}\cdot L, & i\leq n/2 \end{array}$$ (3)

Among them, X_p_ represents the optimal position of the current discoverer. X_worst_ denotes the worst global position currently. A indicates a 1×d matrix, and each of its elements randomly takes the value of 1 or -1. A^+^ satisfies the equation A^+^ = A^T^(AA^T^)^-1^. When i > n / 2, it represents that the fitness value of the i-th follower is poor, has not obtained food, is in a state of starvation, and needs to fly to other areas to forage. When i ≤ n / 2, the follower follows the discoverer who is in the optimal position at this time to forage.

A certain number of sparrows are randomly chosen from the population to be responsible for vigilance (watchers). If danger approaches, the watchers will immediately abandon their food and move to a new location to continue foraging while sounding an alarm signal. The position update formula for the watchers can be described by the following equation:

$$X_{i}^{t+1}=\{\begin{array}{ll} X_{best}^{t}+\beta \cdot |X_{i}^{t}-X_{best}^{t}|, & f_{i}>f_{g}\\ X_{i}^{t}+K\cdot (\frac{\left|X_{i}^{t}-X_{worst}^{t}\right|}{(f_{i}+f_{\omega })+\varepsilon }), & f_{i}=f_{g} \end{array}$$ (4)

Where X_best_ indicates the best global position currently. β and K are both step control parameters. β denotes a random number that follows a standard normal distribution, and K represents a random number in the range of [–1, 1]. f_i_ indicates the fitness value of the current watcher. f_g_ and f_ω_ denote the best and worst fitness values in the present entire sparrow population, respectively. Ɛ is a very small constant to avoid the denominator being 0. When f_i_ > f_g_, it represents that the current watcher is on the boundary of the population and is easy to become the target of predators. When f_i_ = f_g_, it indicates that the watcher in the middle of the population has sensed danger and needs to approach other companions to ensure its own safety.

### 4.2 Improved strategies

#### 4.2.1 Good point set theory

Research has shown that the distribution of the initial population has a crucial impact on the search results of swarm intelligence optimization algorithms [31]. However, the sparrow search algorithm uses the way of random sampling to generate the initial population. This can lead to an uneven distribution of sparrows in the search space and a lack of diversity, thereby affecting the optimization ability of the algorithm. At the same time, during multiple experiments, the inconsistency in the initial population distribution leads to differences in the results of the algorithm’s operation. The stability is poor. To address these issues, this paper introduces the good point set theory [32, 33]. The population initialized by the good point set can be evenly distributed in the search space, effectively improving the diversity of the sparrows. The stability of the good point set is high, and the spatial distribution obtained by solving the good points multiple times is consistent. Assuming the number of sparrows is n, and the dimension of the search space is d, then the method of initializing the population with a good point set is as follows:

$$P(k)=\{(r_{1}*k,r_{2}*k,\cdots ,r_{d}*k),k=1,2,\cdots ,n\}$$ (5)

Among them, *r*_*i*_ = 2cos(2*πi*/*p*), p is the smallest prime number that satisfies the inequality (*p*−3)/2≥*d*. The following formula can map *P*(*k*) to the search space where the population is located:

$$X_{k}^{j}=a_{j}+\mathrm{mod}(P{\left(k\right)}^{j},1)*(b_{j}-a_{j})$$ (6)

In the formula, $X_{k}^{j}$ represents the position of the kth sparrow in the jth dimension. a_j_ and b_j_ respectively denote the lower and upper limits of the jth dimension.

**Fig 1:**
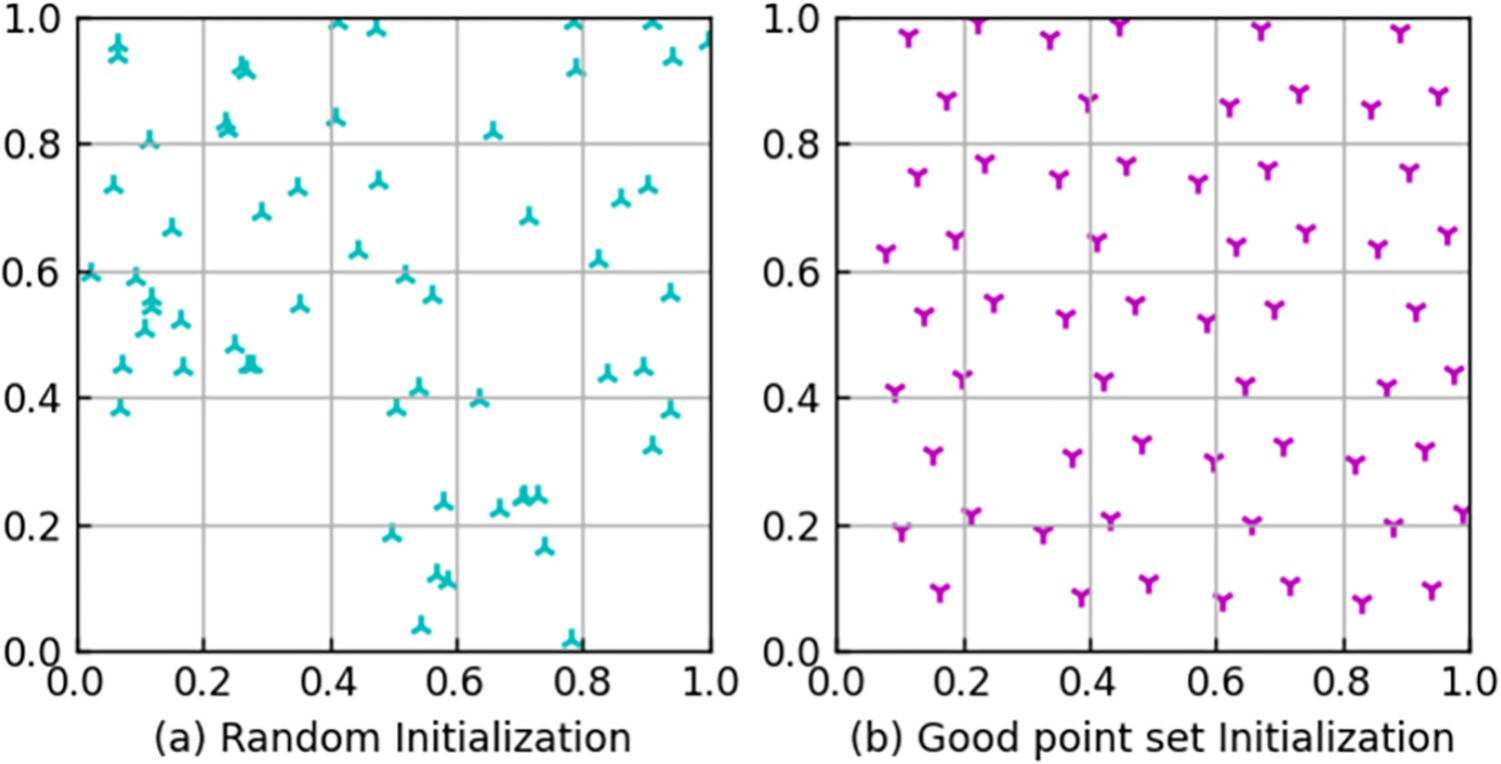
Comparison of the initial population. (a) Random Initialization. (b) Good point set Initialization.

Fig 1(A) is the initial population generated by random initialization. Fig 1(B) is the initial population generated by good point set initialization. By comparison, it can be found that the effect of initializing the population with a good point set is better.

#### 4.2.2 Proportion-Adaptive strategy

According to the foraging mechanism of the sparrow, it is known that the discoverers and followers are responsible for global search and local search, respectively. However, since the proportion of discoverers in the population is constant, this will cause the algorithm to be unable to balance the two search methods. In the early stage of the algorithm, the search space is large, and the number of discoverers is small, which cannot carry out sufficient global search. In the later stage of the algorithm, the search area gradually decreases, the number of discoverers is larger, and the number of followers is smaller, which cannot carry out sufficient local search. To this end, this paper addressed the above problem by dynamically adjusting the percentage of discoverers in the population. The formula is as follows:

$$P=\min\_+(\max\_-\min\_)*\cos(\frac{\pi }{2}*{\left(\frac{t}{T}\right)}^{2})$$ (7)

**Fig 2:**
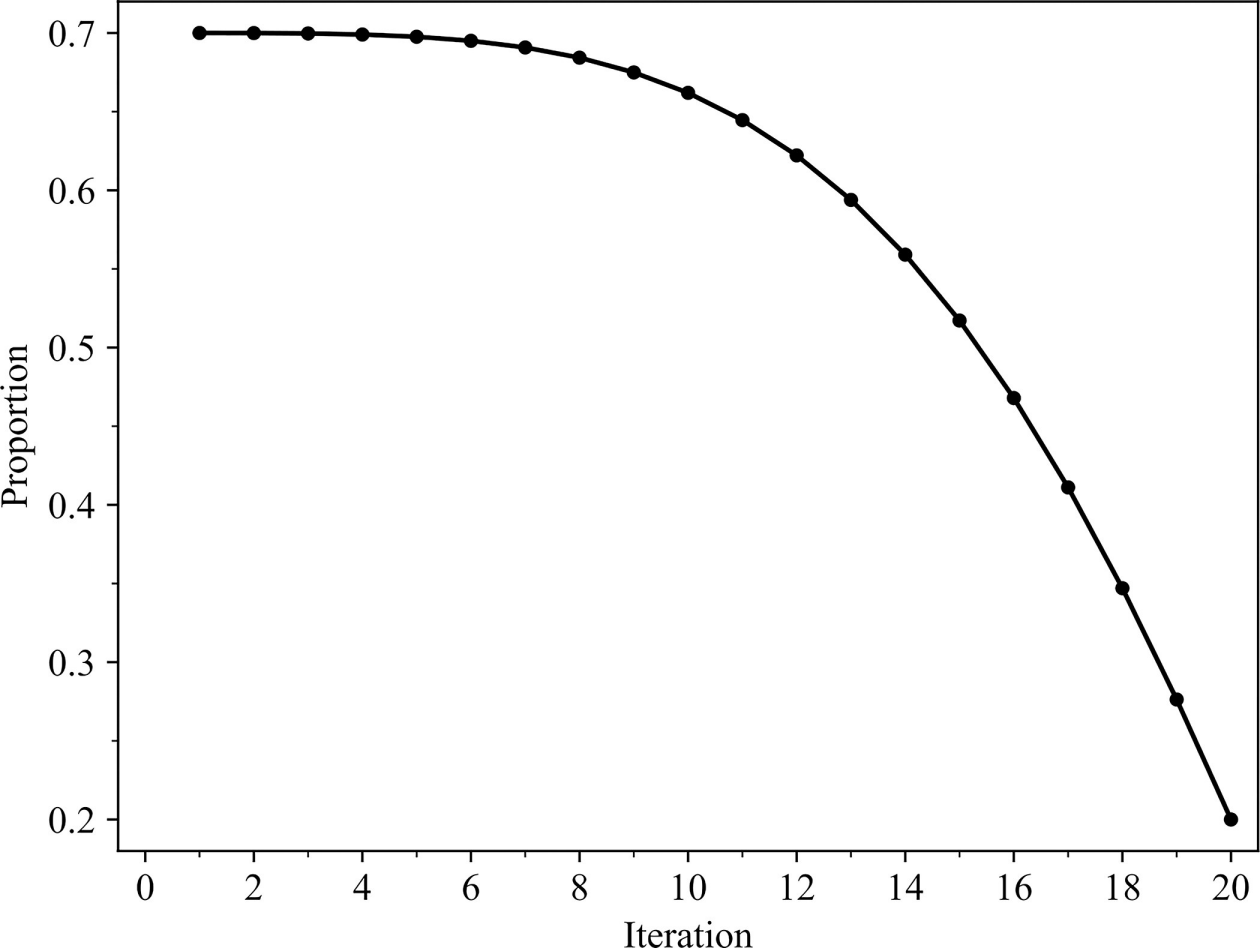
Dynamic change diagram of the proportion of discoverer sparrows.

Where max_ and min_ represent the maximum and minimum values of the proportion of the population occupied by the discoverer, respectively. t is the current number of iterations. T is a constant, denoting the total number of iterations. When max_ = 0.7, min_ = 0.2, and T = 20, the change in the proportion of the population occupied by the discoverer sparrow is shown in Fig 2.

Fig 2 shows the changes in the proportion of discoverers throughout the entire iteration process. It can be observed that in the early stage, the proportion of discoverers is larger, which allows for sufficient global search. In the later stage, the proportion of discoverers gradually decreases, the proportion of followers increases, and the local search capability is enhanced.

#### 4.2.3 Improved location update method

In the sparrow search algorithm, if the alarm value is less than the safety value, the position update formula for the discoverers is:

$$X_{i}^{t+1}=X_{i}^{t}\cdot \exp(-\frac{i}{\alpha \cdot T})$$ (8)

The updated position is the present position multiplied by the exponential function with the natural logarithm as the base. Through analysis, it can be found that the search range of the discoverer is gradually decreasing and tending to zero. This will affect the convergence speed of the algorithm and make the algorithm prone to local optimum. To address this issue, this paper has modified the position update formula for the discoverers, as shown in the following equation:

$$X_{i}^{t+1}=\{\begin{array}{ll} X_{i}^{t}\cdot (1+Q), & R_{2}<ST\\ X_{i}^{t}+Q\cdot L, & R_{2}\geq ST \end{array}$$ (9)

This paper improved the traditional SSA through the above three strategies and named it the multi-strategy modified sparrow search algorithm (MSMSSA). The implementation procedure of MSMSSA is illustrated in Algorithm 1.

**Algorithm 1:** The framework of the MSMSSA.

**Input:**

T: the total number of iterations

max_: the maximum proportion of discoverers in the population

min_: the minimum proportion of discoverers in the population

number: the number of sentinels

ST: the safety value

Initializing a population of n sparrows using the good point set method

Establish a fitness function *F*(*X*), where *X* = (*x*_1_,*x*_2_,⋯*x*_*d*_)

**Output:** *X*_*best*_, *F*_*g*_

Calculate fitness values and sort them, recording the current best and worst individuals

**while** (t ≤ T)

    Calculate P

    *R*_2_ = rand(1)

    **for** i = 1 to P*n **do**

        Update the location of discoverers according to Eq (9)

    **end for**

    **for** i = P*n+1 to n **do**

        Update the location of followers according to Eq (3)

    **end for**

    **for** i = 1 to number **do**

        Update the location of sentinels according to Eq (4)

    **end for**

    Calculate the fitness values of the new location and update if it is better

    t = t + 1

**end while**

**return** *X*_*best*_, *F*_*g*_

### 4.3 MSMSSA-LSTM model

The real target of cross-variety arbitrage trading is the spread between different futures contracts. When the spread is higher than the equilibrium state, a short strategy is adopted, and when it is lower than the equilibrium state, a long strategy is adopted. Profits can be obtained through the regression of spread. Therefore, in order to seek higher returns, it is particularly important to accurately predict the spread. Since the LSTM model performs excellently in dealing with time series problems, this paper builds a spread prediction model for futures data based on it. Selecting appropriate hyperparameters in LSTM can effectively improve the topology of the network model and enhance its fitting and generalization capabilities. As a consequence, to match the network model structure with the characteristics of futures data, this paper combines the MSMSSA algorithm with the LSTM model to construct a MSMSSA-LSTM prediction model.

#### 4.3.1 Structure of MSMSSA-LSTM

In the MSMSSA-LSTM model, the network structure of LSTM mainly includes the input layer, LSTM layer, Dense layer, and output layer. The objective of the MSMSSA algorithm optimization is the learning rate, the number of model training, the number of neurons in two hidden layers, and the size of the time window, which are five hyperparameters. Based on the parameters set in advance, the first generation of the sparrow population is generated using the method of initializing with a good point set, and LSTM modeling is carried out for individuals in the population in turn. The mean square error (MSE) of the model on the validation set is used as the fitness function of the MSMSSA algorithm, and its calculation formula is as follows:

$$f=\frac{1}{n}\sum_{i=1}^{n}{\left({\hat{y}}_{i}-y_{i}\right)}^{2}$$ (10)

Among them, n is the sample size of the validation set. ${\hat{y}}_{i}$ and *y*_*i*_ respectively represent the predicted value and the actual value of the i-th sample.

**Fig 3:**
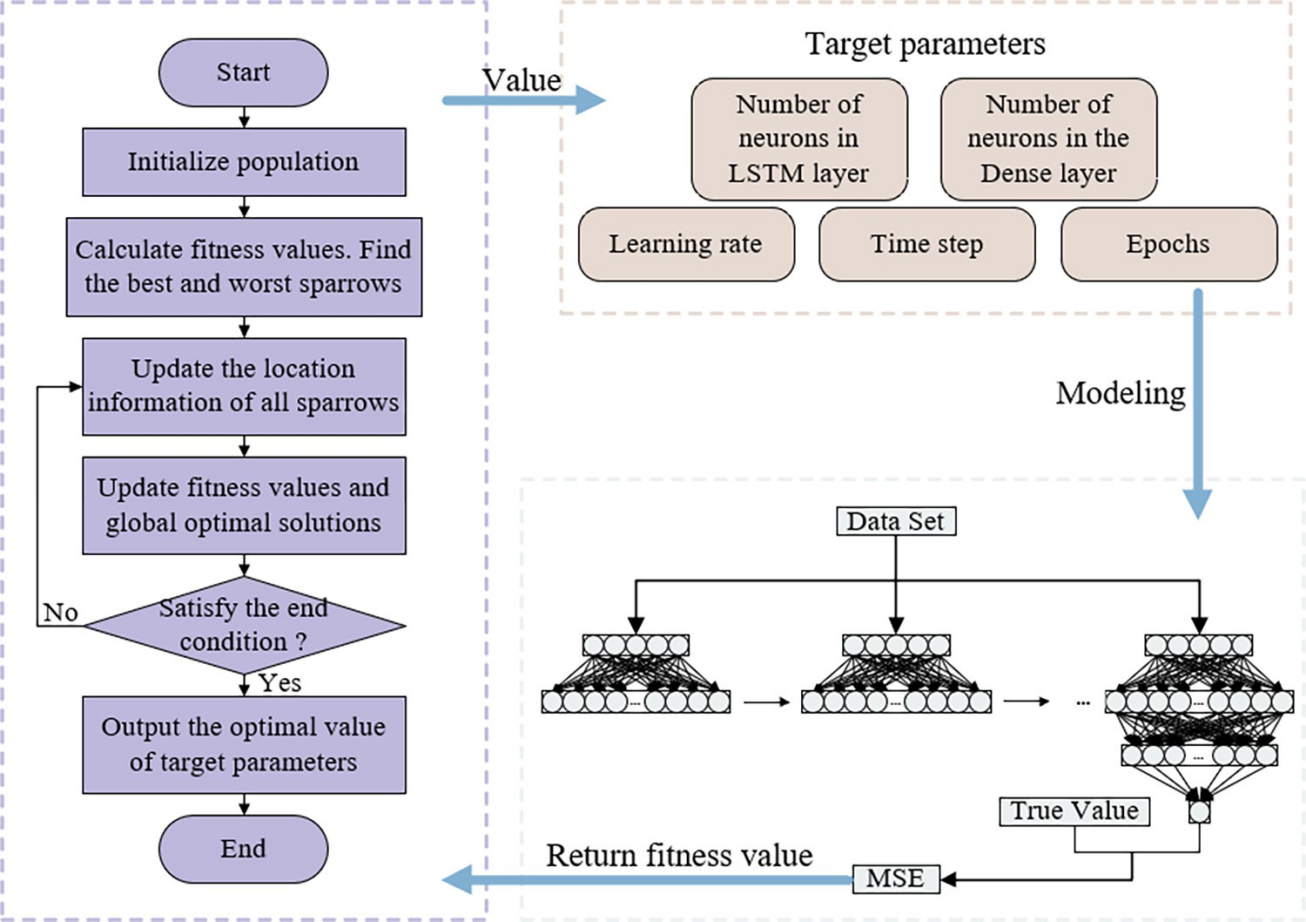
The structure of the MSMSSA-LSTM model.

According to the fitness values of each individual in the sparrow population, they are divided into discoverers and followers, and their positions are updated by Formulas (9) and (3). A certain number of vigilant sparrows are randomly selected and updated according to Formula (4). It is judged whether the termination condition is satisfied. If it is satisfied, the optimal value of the target parameter is output. Otherwise, it is re-divided. Continue to update position information and calculate fitness values until the termination condition is satisfied. Finally, the LSTM model is constructed based on the obtained optimal value of the target parameter to realize arbitrage spread prediction. The structure of the MSMSSA-LSTM model is shown in Fig 3.

#### 4.3.2 Algorithm flow of MSMSSA-LSTM

The specific steps for optimizing LSTM network hyperparameters using the MSMSSA algorithm are as follows:

*Step 1*. Process the data. Determine the input features of the model. Check whether the data is missing, abnormal, disordered, etc. If it exists, process the data through corresponding preprocessing operations. Normalize the data. Divide the data into training sets, validation sets, and test sets according to a certain proportion.

*Step 2*. Set the parameters. Set the parameters in the MSMSSA algorithm, such as population size, number of iterations, maximum and minimum proportions of the discoverer sparrow in the population, number of watchers, safety values, etc.

*Step 3*. Generate the initial population. Based on parameters such as the number of populations, the dimension of the search space, the upper and lower limits of each target parameter value, etc., generate the initial population through the method of initializing the good point set.

*Step 4*. Calculate the fitness value. Perform LSTM modeling according to the target parameters corresponding to each sparrow, return the mean square error on the validation set as its fitness value, sort the fitness values, and find out the best and worst sparrow individuals.

*Step 5*. Update the location information. Calculate the number of discoverers, followers, and watchers, update their location information according to Formulas (9), (3)~(4), compare the global optimal solution, and update the optimal fitness value.

*Step 6*. Determine the termination condition. When the number of iterations reaches the maximum, return the optimal value of the target parameters. Otherwise, go back to step 4 and continue execution.

*Step 7*. Build the model. Build the LSTM model according to the optimal value of the target parameters.

*Step 8*. Make a prediction. Train the model with the training set and validation set data, use the trained model to predict the test set, and get the prediction results.

**Fig 4:**
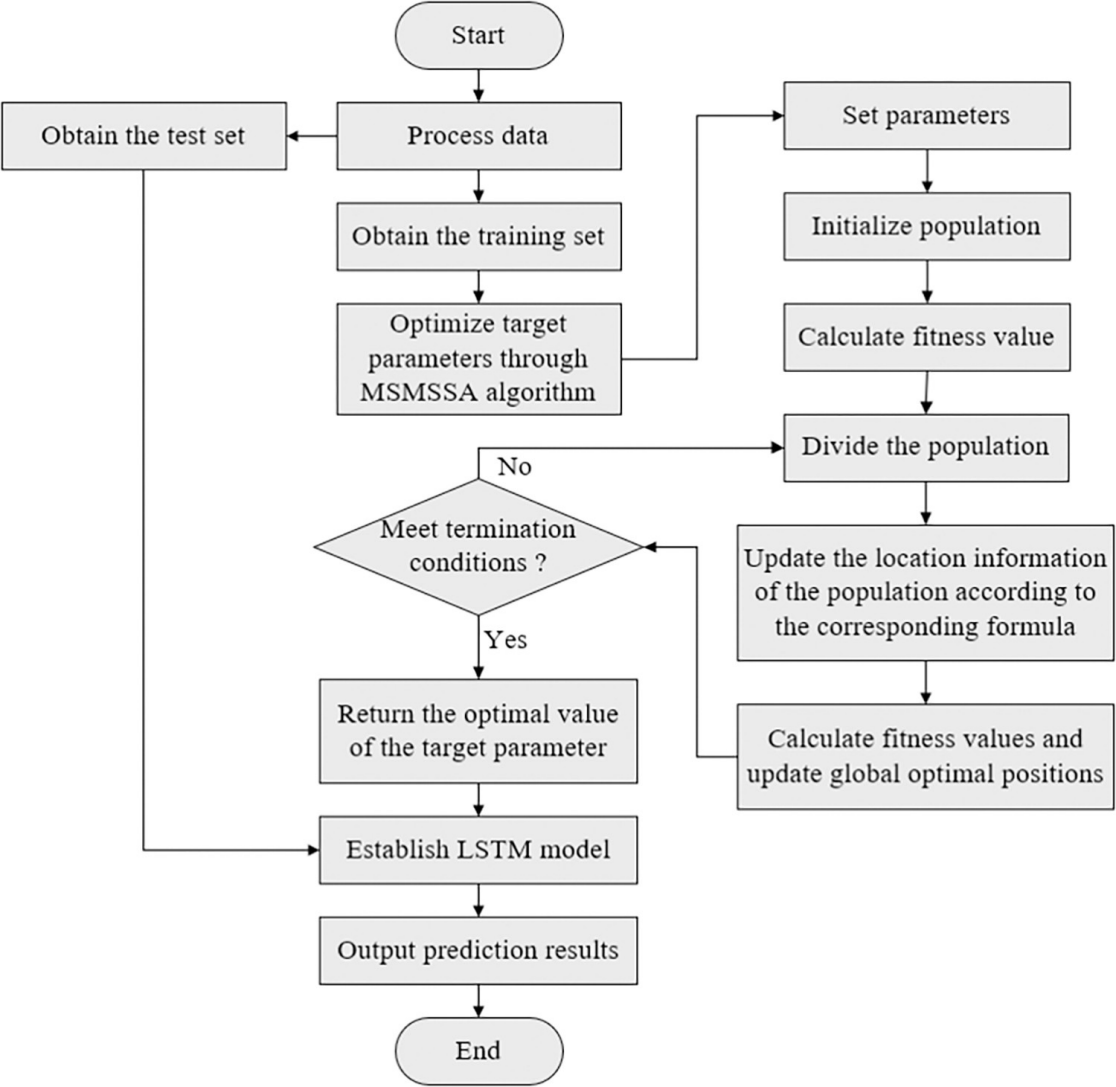
The flowchart of the MSMSSA-LSTM algorithm.

The flowchart of the MSMSSA-LSTM algorithm is shown in Fig 4.

## 5 Experiments

To prove the effectiveness of MSMSSA-LSTM, this paper compared this method with MLP, RNN, LSTM, GRU, and SSA-LSTM using the same training set and test set data under the same operating environment. All the experiments are based on the TensorFlow deep learning framework under the CentOS operating system, configured with NVIDIA CUDA 10.1 and cuDNN 7.6 deep learning libraries to accelerate GPU computing. The Python version is 3.7, and the TensorFlow version is 2.3. According to the influence factors, including the opening price spread, highest price spread, lowest price spread, closing price spread, moving average convergence and divergence (MACD), differential exponential average (DEA), difference (DIF), and price spread fluctuation, the next minute’s closing price spread is predicted.

### 5.1 Data

#### 5.1.1 Data description

**Fig 5:**
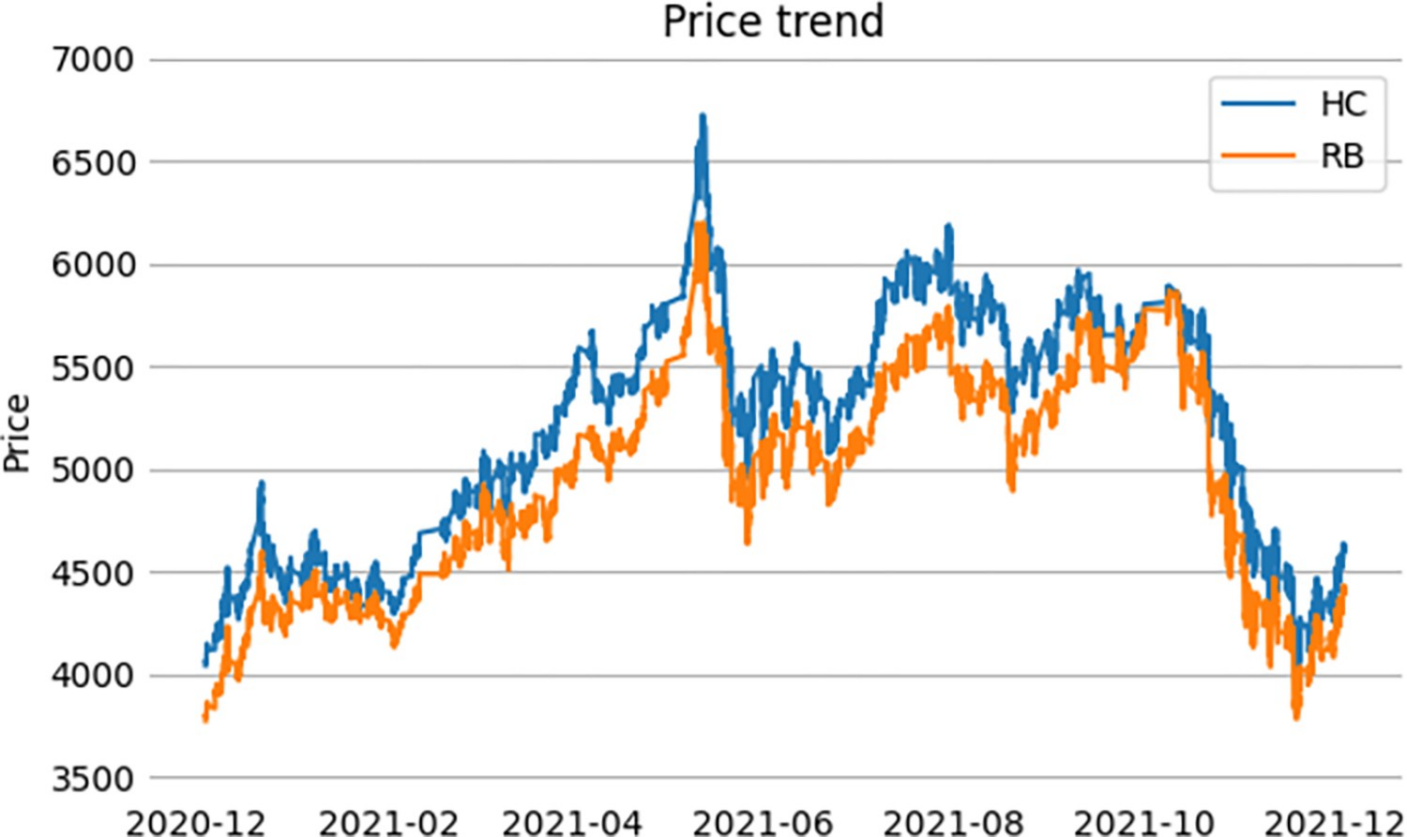
The 1-minute trend of the closing prices of two futures in 2021.

This article selects the main contract data of rebar and hot coil futures from December 4, 2020, to February 16, 2023, as the research object. To ensure the continuity of the data and avoid the impact of contract delisting, another main contract data is used as a replacement when it is two months away from the delivery period. At the same time, to improve the predictive performance of the model, a large amount of data is needed for training. Therefore, this article uses high-frequency trading data of 1-minute prices of rebar and hot coil futures for research, totaling 180000 sets of data. They are divided into training and testing sets in an 8:2 ratio. The training set is mainly used for optimizing target parameters and training the model, while the testing set is mainly used to verify the predictive effect of the model outside of the sample. The data is sourced from Eastern Wealth Choice data. Fig 5 shows the 1-minute trend of the closing prices of two futures in 2021.

As can be visualized from the chart above, the price trends of rebar and hot coil futures are extremely similar. Table 1 shows the result of a basic statistical analysis of the price data for the two futures.

**Table 1:** The results of basic statistical analysis.

Variety	Maximum	Minimum	Average	Skewness	Kurtosis
Hot coil	6723	3452	4742	0.1639	2.0922
Rebar	6198	3392	4570	0.1627	2.1290

#### 5.1.2 Correlation analysis

Arbitrage trading can only be carried out among futures varieties with a strong correlation. The calculation formula for correlation is as follows:

$$\beta_{XY}=\frac{\sum_{i=1}^{n}\left(X_{i}-\bar{X})(Y_{i}-\bar{Y}\right)}{\sqrt{\sum_{i=1}^{n}{\left(X_{i}-\bar{X}\right)}^{2}}\sqrt{\sum_{i=1}^{n}{\left(Y_{i}-\bar{Y}\right)}^{2}}}$$ (11)

Where X and Y denote the price series of two different futures varieties respectively. n is the total number of samples. X_i_ and Y_i_ represent the ith sample in the respective price series. $\bar{X}$ and $\bar{Y}$ are the average values of the respective price series. The correlation between the two futures of rebar and hot coil can be obtained by using Eq (11) as shown in Table 2.

**Table 2:** The correlation coefficient of rebar and hot coil futures.

	Rebar	Hot coil
**Rebar**	1.0000	0.9905
**Hot coil**	0.9905	1.0000

Table 2 shows that the correlation coefficient of rebar and hot coil is 0.9905, indicating that there is a very strong correlation between the two, and arbitrage trading can be constructed. However, the strength of the correlation cannot reflect the stability of the spread between the two futures varieties, so a cointegration test needs to be performed.

#### 5.1.3 Cointegration test

The cointegration test can only be performed if each data series satisfies the same order of single integrality. Therefore, this research needs to carry out the unit root test on the price series of rebar and hot coil futures first to determine their smoothness and the order of single integrality. This research uses Eviews software to conduct an ADF test to get Table 3.

**Table 3:** ADF unit root test.

Variety	ADF test value	1% critical value	5% critical value	10% critical value	P-value	Conclusion
**HC (Hot coil)**	-0.198257	-2.564975	-1.940826	-1.616699	0.6150	Unsteady
**RB (Rebar)**	-0.087496	-2.564975	-1.940826	-1.616699	0.6536	Unsteady
**ρHC**	-309.5277	-2.564975	-1.940826	-1.616699	0.0001	Steady
**ρRB**	-309.3130	-2.564975	-1.940826	-1.616699	0.0001	Steady

As can be seen from the table, the ADF test values of HC and RB are both greater than the critical values at the 1%, 5%, and 10% significance levels, and the P values are all greater than 0.05. Therefore, the price series of hot coil and rebar futures have a unit root and are non-stationary series. On this basis, the first-order difference is performed to obtain the series ρHC and ρRB. The ADF test values are all less than the critical values at the 1%, 5%, and 10% significance levels, and the P values are all less than 0.05. Therefore, there is no unit root and it is a stationary series. Therefore, the price series of these two futures varieties are both first-order integrated, meet the cointegration conditions, and can undergo cointegration testing.

This paper further tests the cointegration relationship between the price series of hot coil and rebar futures using the EG two-step method. First, the OLS method is used to obtain the following cointegration model:

$$HC=-459.8848+1.138095RB+\varepsilon_{t0}$$ (12)

Among them, HC is the explained variable and RB is the explanatory variable. Ɛ_t0_ is random error. In this case, the value of R^2^ is 0.981099, which indicates that the model has a 98.1% probability of explaining the real situation well, and the fitting effect is good. Next, the smoothness of the residual series is tested by the ADF method and the results are shown in Table 4.

**Table 4:** Results of residual series stationarity test.

Residual series	ADF test value	1% critical value	5% critical value	10% critical value
e_t_	-4.377162	-3.900159	-3.337733	-3.046223

Table 4 illustrates that at the 1% significance level, the ADF test value of the residual series is less than the critical value, that is, the null hypothesis is rejected and the series is considered to be stationary. According to EG cointegration theory, the price series of hot coil and rebar futures have a stable long-term equilibrium relationship.

### 5.2 Evaluation criteria

To quantitatively measure the prediction effect of each model, this paper uses the mean absolute percentage error (MAPE), the root mean square error (RMSE), the mean absolute error (MAE), and the coefficient of determination (R^2^) as evaluation indexes. If the corresponding values of MAPE, RMSE, and MAE are smaller, and R^2^ is closer to 1, it indicates that the prediction effect of the model is better. The specific calculation formula is as follows:

$$MAPE=\frac{1}{n}\sum_{i=1}^{n}\left|\frac{{\hat{y}}_{i}-y_{i}}{y_{i}}\right|$$ (13)

$$RMSE=\sqrt{\frac{1}{n}\sum_{i=1}^{n}{\left({\hat{y}}_{i}-y_{i}\right)}^{2}}$$ (14)

$$MAE=\frac{1}{n}\sum_{i=1}^{n}\left|{\hat{y}}_{i}-y_{i}\right|$$ (15)

$$R^{2}=\frac{\sum_{i=1}^{n}{\left({\hat{y}}_{i}-\bar{y}\right)}^{2}}{\sum_{i=1}^{n}{\left(y_{i}-\bar{y}\right)}^{2}}$$ (16)

Among them, *n* represents the total number of samples in the test set. ${\hat{y}}_{i}$ and *y*_*i*_ denote the predicted and true values of the model, respectively. $\bar{y}$ indicates the average of all true values.

### 5.3 Model implementation

#### 5.3.1 Initialization of parameters

In the MSMSSA-LSTM model, this research chooses the mean square error MSE as the loss function, uses the Adam algorithm as the optimizer, and sets Dropout to 0.1 to prevent overfitting. The number of sparrows in the population is 15, in which the maximum value of the proportion of discoverers is 0.7 and the minimum value is 0.2. The safe value is 0.8. The percentage of watchers is set to 20%. The number of iterations of the algorithm is 20. There are five objective parameters to be optimized by the MSMSSA algorithm, which are the learning rate, the number of iterations, the number of neurons in the two hidden layers, and the time step. Before starting the optimization, each objective parameter should be limited to a reasonable search range to avoid the waste of resources. In this paper, combined with relevant references and existing research [34–36], the appropriate search range of target parameters is finally selected, as shown in Table 5.

**Table 5:** The search range for target parameters.

Target parameters	Search range
Learning rate	[0.001, 0.01]
Epoch	[10, 100]
Neuron numbers in the first hidden layer	[1, 100]
Neuron numbers in the second hidden layer	[1, 100]
Time step	[5, 50]

#### 5.3.2 Comparison of algorithms

To verify the performance of the improved sparrow search algorithm, the SSA-LSTM model was designed for comparison. Meanwhile, in order to avoid deviations in the results of a single run, this study conducted 10 independent experiments on MSMSSA-LSTM and SSA-LSTM, respectively. During the experiments, the same parameter settings were used for both methods. Specific details can be found in 5.3.1. Table 6 shows the optimization results of the objective function.

**Table 6:** The optimization results of the objective function.

Algorithm	Best	Worst	Mean	Median	STD	Variance
SSA	1.6807e-04	1.8595e-04	1.7654e-04	1.7501e-04	6.0240e-06	3.6288e-11
MSMSSA	1.6646e-04	1.7667e-04	1.7041e-04	1.6915e-04	3.9855e-06	1.5884e-11

It can be found that the best value, the worst value, the average value, and the median value of the optimization results of the MSMSSA algorithm are better than those of the SSA algorithm, which indicates that MSMSSA has stronger spatial search capability and higher convergence accuracy. The standard deviation and variance of the MSMSSA algorithm are also smaller than the SSA algorithm, indicating that MSMSSA has higher stability.

**Fig 6:**
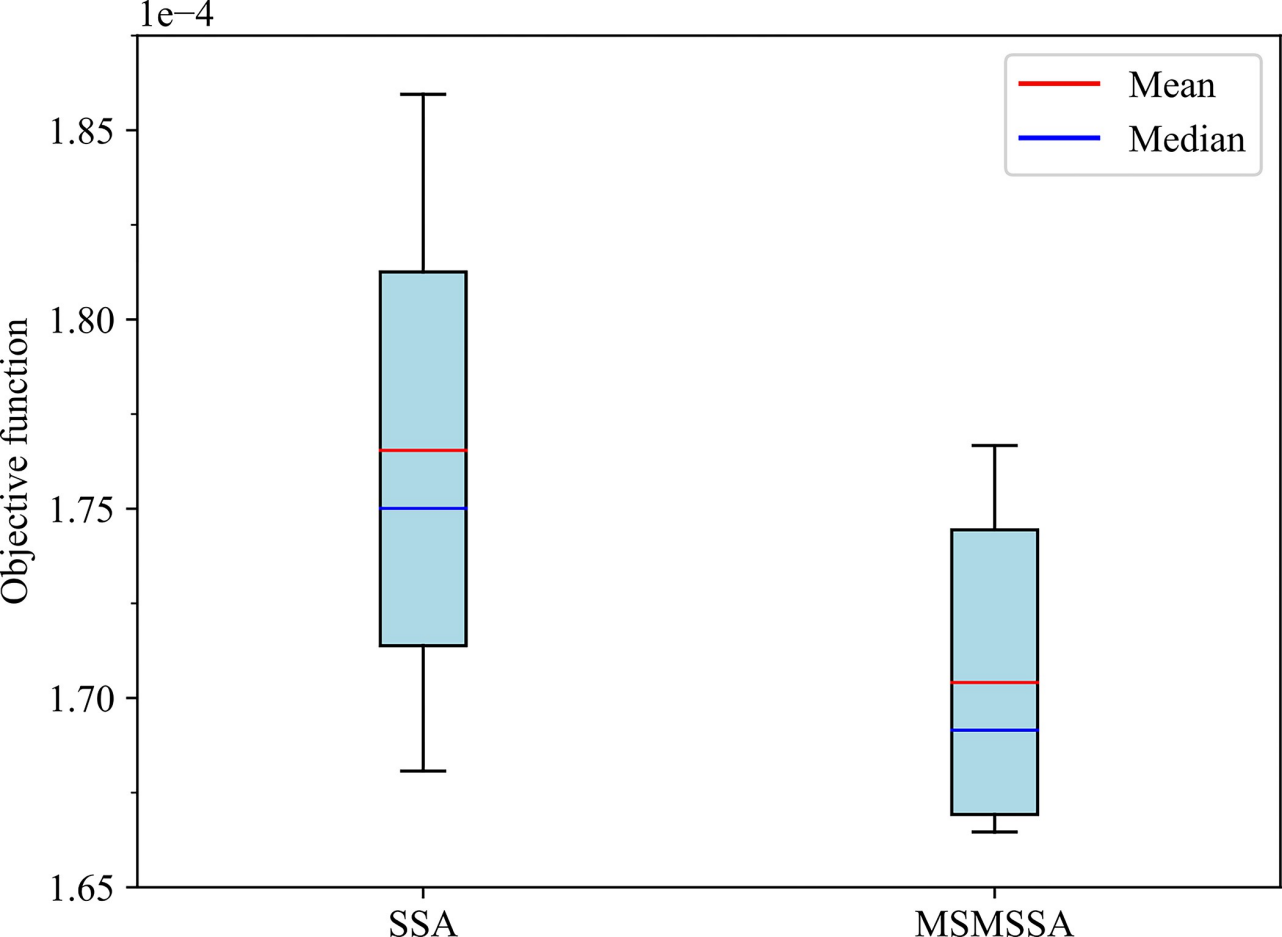
Box-plot of the optimization results of the two algorithms.

In addition, this research also plots the optimization results of the two algorithms on the objective function into a box plot. As shown in Fig 6. It is not difficult to see that the median and mean of box-plot produced by the MSMSSA algorithm are smaller than those of the SSA algorithm. Therefore, the box-plot of the MSMSSA algorithm is in a lower position, which indicates that the overall quality of the solution generated by the MSMSSA algorithm is better than that of the SSA algorithm. At the same time, the IQR of the box-plot generated by the MSMSSA algorithm and the SSA algorithm are 8.91e-06 and 1.178e-05, respectively, indicating that the MSMSSA algorithm produces smaller discrete degrees and more stable optimization results.

**Fig 7:**
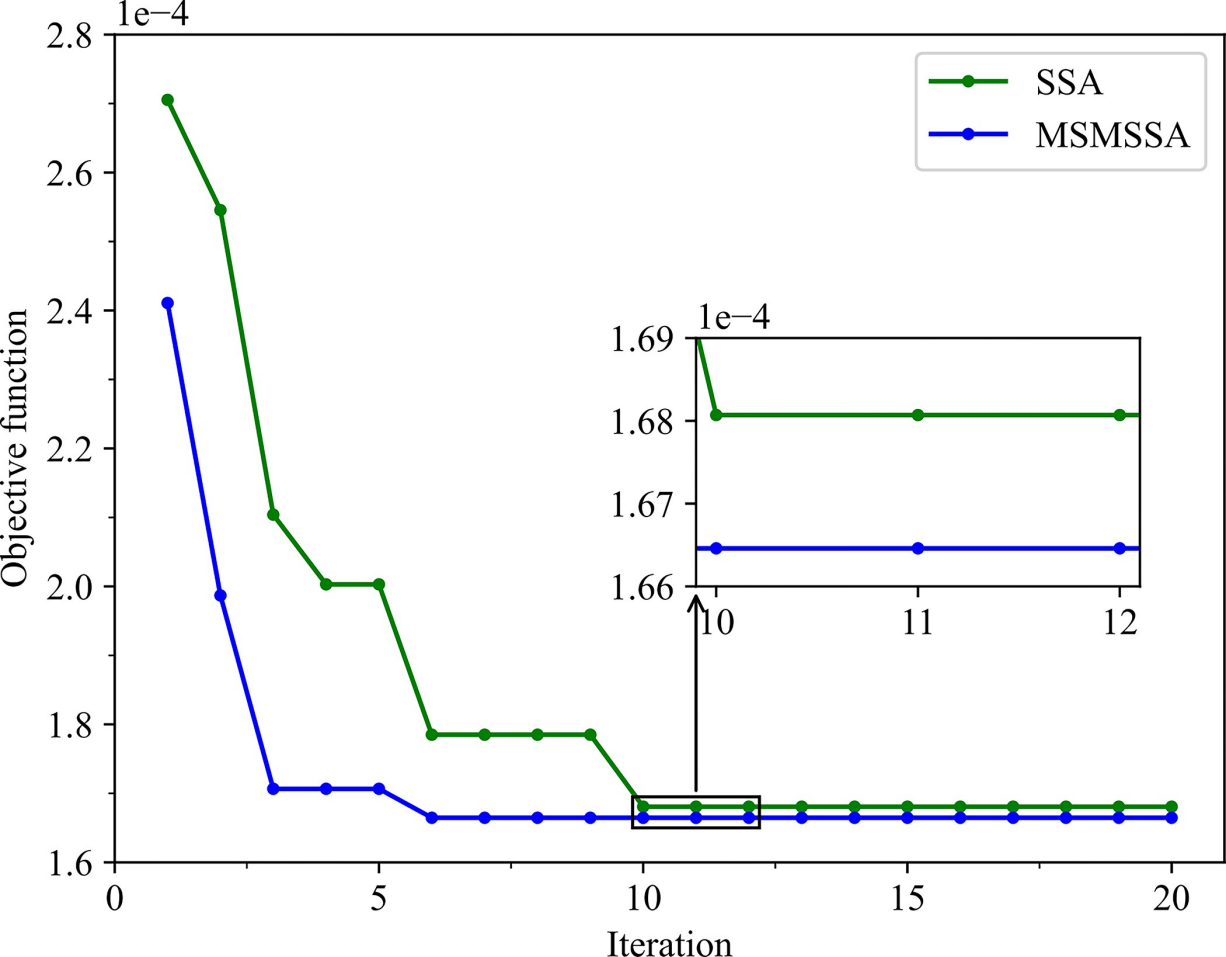
Convergence curves of the best optimization results of the two algorithms.

Fig 7 shows the convergence curves of the best optimization results of the two algorithms. The MSMSSA algorithm found the minimum value of the objective function 1.6646e-04 at the 6th iteration. The SSA algorithm found the optimal objective function value at the 10th iteration, which is 1.6807e-04. Therefore, the MSMSSA algorithm outperforms the SSA algorithm both in terms of convergence speed and optimization accuracy.

According to the above analysis, the MSMSSA algorithm has stronger space exploration performance, more accurate optimization precision, better robustness, and faster convergence speed than the standard sparrow search algorithm. This indicates that the MSMSSA algorithm can find a better combination of hyperparameters in the LSTM model, and provide help for constructing high-precision arbitrage spread prediction model.

#### 5.3.3 Optimization of target parameters

**Fig 8:**
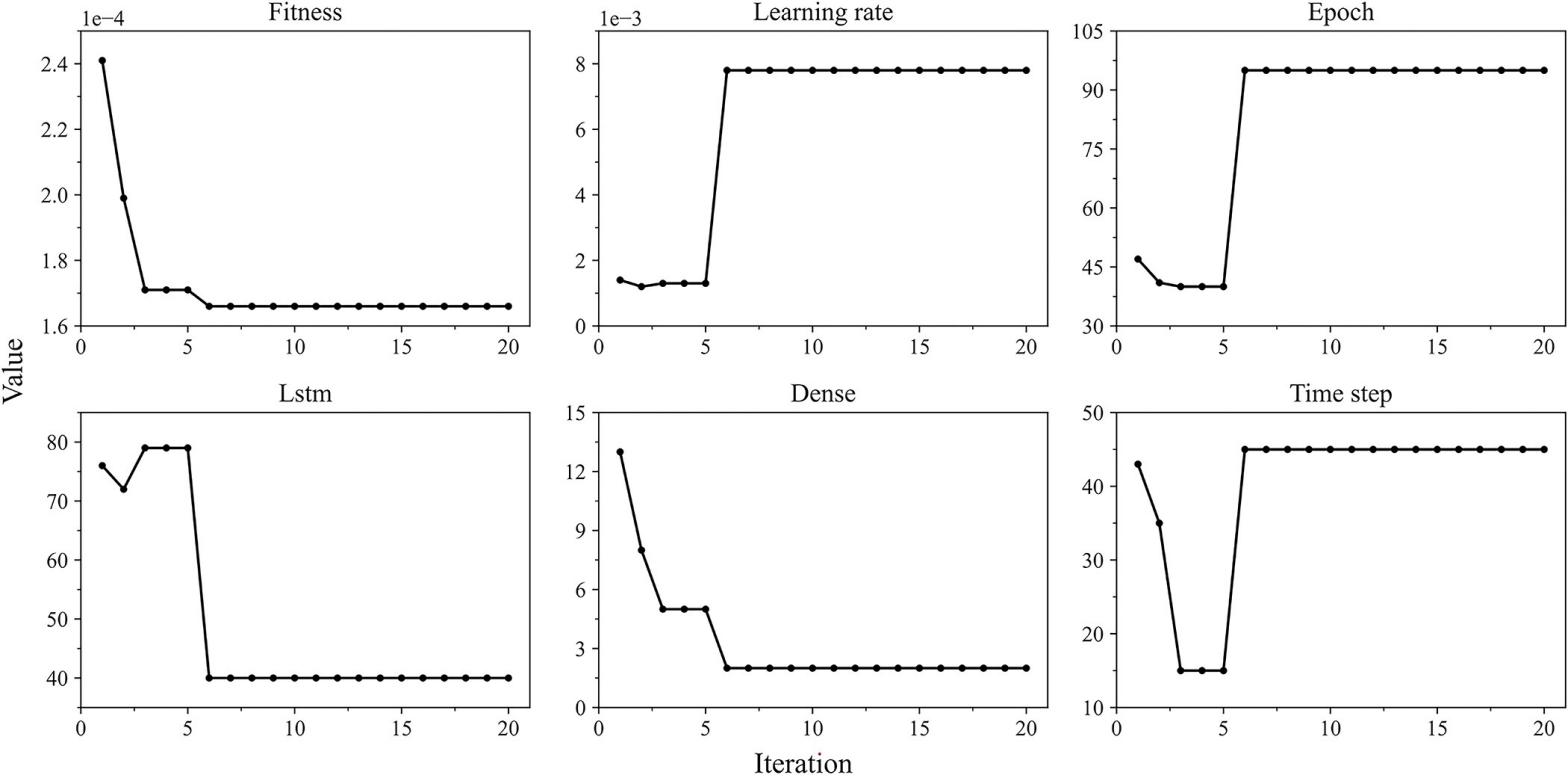
Optimization iterative process of target parameters.

In 10 independent experiments, when the objective function achieves the minimum value, the changes of each parameter during the optimization process are shown in Fig 8 and Table 7. It is not difficult to find that when the MSMSSA algorithm is executed to the 6th round, the fitness value is 0.00016646. At the same time, this value remains stable in the subsequent iteration process and no longer changes, indicating that the optimal parameter combination in the model has been found, that is, the learning rate is 0.00775587, the number of epochs is 95, the number of neurons in the LSTM layer is 40, the number of neurons in the Dense layer is 2, and the time step is 45. This paper constructs a high-precision LSTM model to achieve arbitrage spread prediction through the optimal value of these parameters.

**Table 7:** Optimization iterative process of target parameters.

Iterative process	Fitness value	Learning rate	Epoch	Neuron numbers in the first hidden layer	Neuron numbers in the second hidden layer	Time step
1	0.00024109	0.00139690	47	76	13	43
2	0.00019869	0.00117265	41	72	8	35
3-5	0.00017066	0.00131146	40	79	5	15
6-20	0.00016646	0.00775587	95	40	2	45

### 5.4 Experimental results and analysis

**Fig 9:**
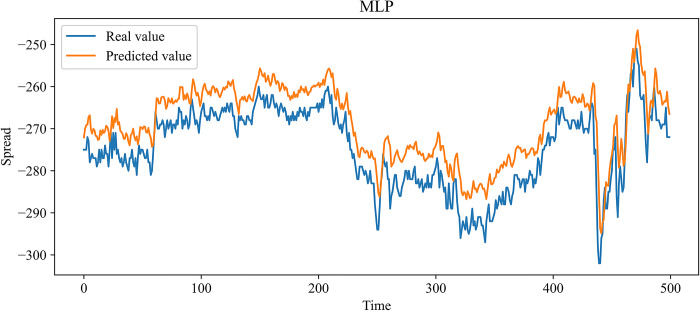
Comparison of the predicted value and the real value for MLP.

**Fig 10:**
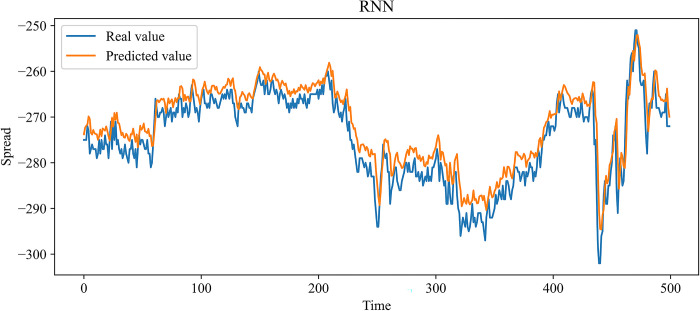
Comparison of the predicted value and the real value for RNN.

**Fig 11:**
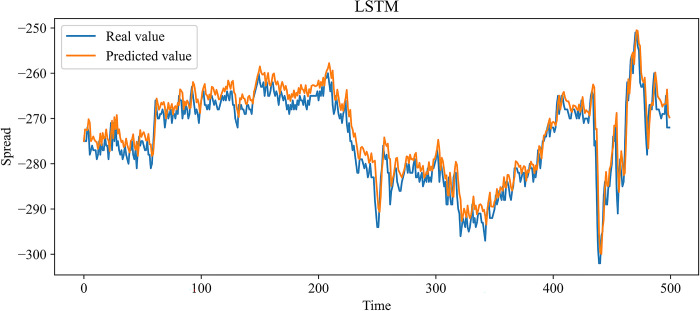
Comparison of the predicted value and the real value for LSTM.

**Fig 12:**
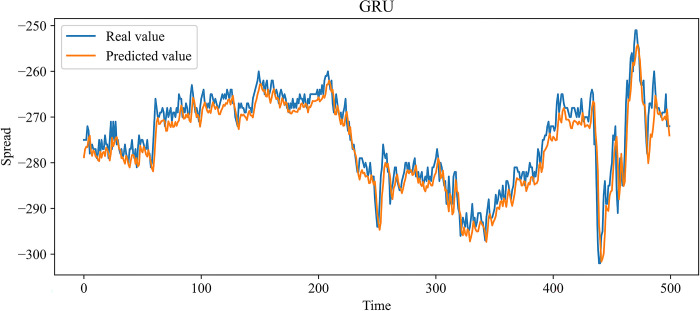
Comparison of the predicted value and the real value for GRU.

**Fig 13:**
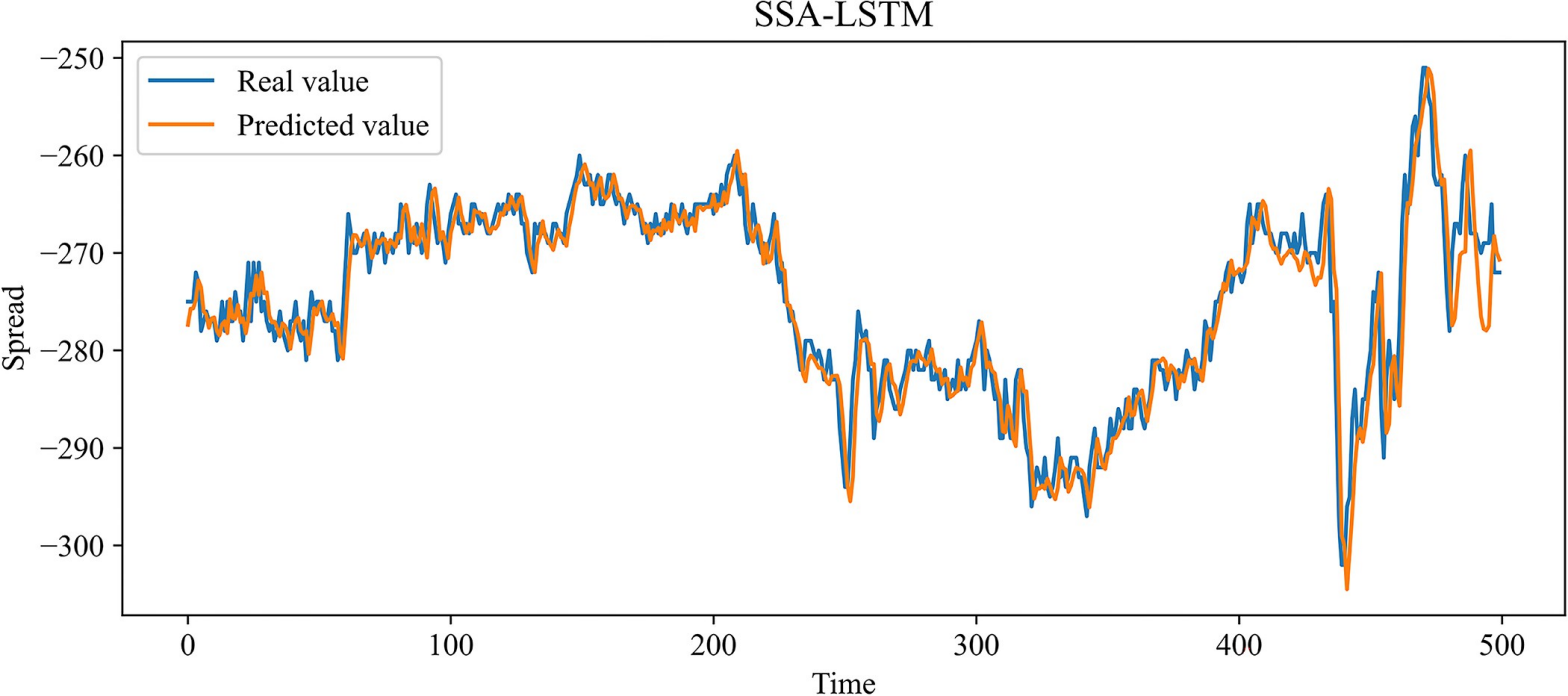
Comparison of the predicted value and the real value for SSA-LSTM.

**Fig 14:**
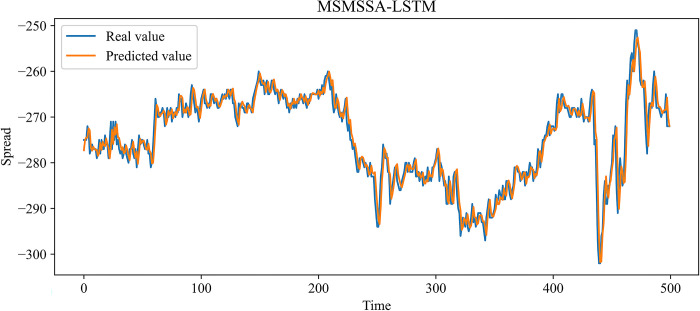
Comparison of the predicted value and the real value for MSMSSA-LSTM.

In this paper, MLP, RNN, LSTM, and GRU, which are more widely used time series forecasting models in the financial field, are chosen as contrast experiments. At the same time, to demonstrate the effectiveness of our improvement on the SSA, an SSA-LSTM model was designed for validation. In the previous 10 independent experiments, the optimal combination of parameters searched by the SSA is as follows: the learning rate is 0.00827520, the number of model training is 48, the number of neurons in the first and second hidden layers is 70 and 3, and the time step is 24. The same training set data is used to train each model, and the test set data is predicted based on the trained model. Figs 9–14 show the prediction results.

In Figs 9–14, among the six forecasting models, the broken line fitting degree of real value and predicted value is MSMSSA-LSTM, SSA-LSTM, GRU, LSTM, RNN, MLP from high to low. Among them, MSMSSA-LSTM has the highest degree of broken line fitting which almost coincides with each other, and MLP has the lowest degree of broken line fitting.

In order to more intuitively reflect the predictive performance of various models on futures spread data and to demonstrate the effectiveness and superiority of the MSMSSA-LSTM model, this paper calculated the evaluation indicators for each model based on their predicted values and real value. The results are shown in Table 8.

**Table 8:** Comparison of six models evaluation indexes.

Model	MAPE	RMSE	MAE	R^2^
MLP	0.0212	7.5799	5.6275	0.9918
RNN	0.0148	4.7028	3.4576	0.9968
LSTM	0.0109	3.3225	2.4217	0.9984
GRU	0.0115	3.2126	2.3824	0.9985
SSA-LSTM	0.0095	2.8525	1.9701	0.9988
MSMSSA-LSTM	0.0088	2.6409	1.8251	0.9990

As shown in Table 8, the MLP model has the largest MAPE, RMSE, and MAE values of 0.0212, 7.5799, and 5.6275, respectively, and the smallest R^2^ value of 0.9918, indicating that the model is hard to fit effectively to futures spread data and has poor predictive performance. Compared with MLP, the predictive performance of RNN has been improved, with MAPE, RMSE, MAE, and R^2^ being 0.0148, 4.7028, 3.4576, and 0.9968, respectively. However, due to the defects of gradient vanishing, gradient explosion, and insufficient long-term memory ability in RNN models, there is still plenty of room for improvement in their prediction accuracy. LSTM effectively solves the problems of the RNN model by introducing gate structure and significantly improves the predictive performance. The four evaluation indicators are, in order, 0.0109, 3.3225, 2.4217, and 0.9984. As a variant of the LSTM model, GRU has MAPE, RMSE, MAE, and R^2^ values of 0.0115, 3.2126, 2.3824, and 0.9985, respectively. From the evaluation indicators, GRU is slightly better than LSTM. The SSA-LSTM model reduces prediction error by using the traditional sparrow search algorithm to find the optimal hyperparameter combination in the LSTM network, with evaluation indicators of 0.0095, 2.8525, 1.9701, and 0.9988, respectively. This paper constructs a MSMSSA-LSTM model by improving the SSA algorithm. Its MAPE, RMSE, and MAE are the smallest, at 0.0088, 2.6409, and 1.8251 respectively, and its R^2^ is the largest, at 0.9990. Compared with the other five models, the MAPE of MSMSSA-LSTM decreased by 58.5%, 40.5%, 19.3%, 23.5%, and 7.4%, respectively. The RMSE decreased by 65.2%, 43.8%, 20.5%, 17.8%, and 7.4%, respectively. The MAE decreased by 67.6%, 47.2%, 24.6%, 23.4%, and 7.4%, respectively. The experimental results show that the MSMSSA-LSTM model proposed in this paper has significantly better prediction accuracy than the other five methods, and the effect is the best.

According to the above analysis, the MSMSSA-LSTM model has a good predictive ability for the trend of arbitrage spread. This can help investors formulate more scientific trading strategies and seek higher returns.

## 6 Conclusion

This research proposes a novel technique for the problem of arbitrage spread forecasting named MSMSSA-LSTM. The technique utilizes MSMSSA to automatically seek the optimal combination of hyperparameters in the LSTM model. This effectively solves the problem that hyperparameters in LSTM are difficult to determine and cannot be adjusted with training. Based on the standard sparrow search algorithm, MSMSSA introduces the good point set theory, the proportion-adaptive strategy, and the improved location update method to further enhance the spatial search capability of SSA. This paper innovatively applies the MSMSSA algorithm and LSTM model to the field of futures arbitrage and has achieved good results. The newly proposed model is evaluated using real spread data of rebar and hot coil futures in the Chinese futures market, and compared with the SSA-LSTM model and several classical machine learning methods. The key findings are as follows:

1. Compared with the SSA algorithm, the MSMSSA algorithm has stronger global optimization ability and better robustness. Faced with hyperparameter optimization problems in LSTM models, the MSMSSA algorithm has shown better applicability.
2. Compared with several classical machine learning methods, the mean absolute error of the proposed model is reduced by at least 23.4%. This indicates that using the MSMSSA algorithm to optimize the LSTM network can minimize the influence of human factors and improve the generalization ability and prediction effect of the model.

In summary, the experimental results show that the MSMSSA-LSTM model outperforms all comparative experiments and has the highest accuracy in arbitrage spread trend prediction. The limitation of this model is that the training time is long. During the hyperparameter optimization process, the LSTM network may run hundreds or thousands of times. The time cost is very high. Therefore, future research will continue to accelerate algorithm optimization and improve model performance.
